# Digital and AI-assisted multimodal supportive care, combining physical activity, nutrition, and pain management during chemotherapy for advanced pancreatic cancer patients: study protocol of the European multicenter randomized controlled trial of the RELEVIUM project

**DOI:** 10.1186/s12885-025-14867-6

**Published:** 2025-10-18

**Authors:** Barlo Hillen, Gabrielle Oestreicher, Lisa Schwab, Kira Enders, Perikles Simon, Anneli Elme, Irit Ben-Aharon, Tal Goshenlago, Cindy Neuzillet, Francesco Sclafani, Eva Ester Molina Beltran, Michael Schuster, Hristina Doncheva, Christian Ruckes, Michael Karlecik, Katja Petrowski, David Rosenbaum, Christos Diou, Aristotelis Ballas, Aggeliki Vlachostergiou, Davide Mastricci, Alexander Scherrer, Maximilian Pilz, Jonas Flechsig, Lazaros Apostolidis, Anna-Maria Krooupa, Sai Chintha, Isa Wasswa Musisi, Nefeli Panagiota Tzavara, Athanasios Kakasis, Xenia Hadjikypri, Styliani Karra, Kyriaki Chatzipanagiotou, Ioannis Drivas, Hugo Dacasa, Markus Moehler

**Affiliations:** 1 Institute of Sport Science, Dpt. Sports Medicine, Disease Prevention & Rehabilitation, Johannes Gutenberg University Mainz, Mainz, Germany; 2 Institute of Occupational, Social and Environmental Medicine, University Medical Center, Johannes Gutenberg University Mainz, Mainz, Germany; 3 Department of Medicine 1, University Medical Center of the Johannes Gutenberg University Mainz, Mainz, Germany; 4 Põhja-Eesti Regionaalhaigla, North Estonia Medical Centre, Tallinn, Estonia; 5 Fishman Oncology Center, Rappaport Faculty of Medicine, Rambam Health Care Campus, Haifa, Israel; 6 Saint-Cloud, University Versailles Saint-Quentin, France and Ambroise Paré University Hospital, Institute Curie, Boulogne-Billancourt, France; 7 Hôpital Universitaire de Bruxelles, Institut Jules Bordet - Hôpital Erasme, Université Libre de Bruxelles, Brussels, Belgium; 8 ROSENBAUM CONSULTING Ltd, Sofia, Bulgaria; 9 International Center for Clinical Studies, University Medical Center of the Johannes Gutenberg University Mainz, Mainz, Germany; 10 Harokopio University of Athens, Athens, Greece; 11 EXUS AI Labs, Athens, Greece; 12 Fraunhofer Institute for Industrial Mathematics, Kaiserslautern, Germany; 13 Centre for Research & Technology Hellas, Thessaloniki, Greece; 14 MCS Datalabs, Berlin, Germany; 15 AINIGMA Technologies, Louvain, Germany; 16 Center for Social Innovation LTD, Nicosia, Cyprus; 17 Diadikasia Business Consulting, Athens, Greece; 18 Futuro Perfecto Innovacion SL, Madrid, Spain

**Keywords:** Digital Health, Telemedicine, Supportive Care, Pancreatic Cancer, Physical Activity, Nutrition, Cachexia, Chemotherapy, Sarcopenia, Personalized Medicine

## Abstract

**Background:**

Multimodal care, including nutritional support, physical exercise, and pain management, is essential to address complex therapeutic challenges in advanced pancreatic cancer. There is an increasing prevalence worldwide in pancreatic cancer and the disease is often diagnosed late leading to limited treatment options and poor prognosis. Advanced pancreatic cancer patients experience a wide range of adverse symptoms that affect their quality of life and require comprehensive and interdisciplinary patient care early in treatment. Integrating digital health, particularly through remote monitoring, plays a vital role in addressing the therapeutic challenges and improving data-driven clinical decision-making. Therefore, the European project RELEVIUM examines the effect of a personalized, digitally assisted multimodal supportive care intervention on health-related quality of life in patients with pancreatic cancer aiming to improve early access to multidisciplinary, cost-effective palliative care.

**Methods:**

In cancer centers in Estonia, Israel, and Germany, 132 patients will be randomly assigned in the prospective randomized controlled trial RELEVIUM-RCT into two groups. Both groups receive standard chemotherapy. The control group follows usual care, while the intervention group receives personalized, digitally assisted multimodal support integrated into usual care. The intervention includes guidance and monitoring on pain, nutrition, fatigue, sarcopenia, and physical activity. Patients track their physical activities, nutritional behavior and rate pain and fatigue daily via a smartwatch and mobile app. The physician analyses these longitudinal data on a dashboard and counsels the patients every two weeks during clinical visits, assisted by an interdisciplinary team and digital support system. The primary endpoint of the study is health-related quality of life including factors such as time until a definitive deterioration in selected dimensions (physical functioning and/or appetite loss) assessed at 8 weeks. Secondary endpoints include longitudinal analyses of efficacy related to pain, physical function, nutrition, sarcopenia, and socioeconomic factors.

**Discussion:**

The RELEVIUM-RCT investigates the efficacy of digital health support for individuals with advanced pancreatic cancer in conjunction with conventional treatments in three European countries. Longitudinal data on the interplay of chemotherapy toxicity, fatigue, pain, physical activity, and nutrition will provide valuable extended insights on multimodal pancreatic cancer care. Moreover, this data can help demonstrate the benefits of digital health in clinical decision-making, ultimately contributing to improved quality of care in Europe.

**Trial registration:**

Registry: German Clinical Trials Register; Registration number: DRKS00037143; Date of registration: 5th of June 2025.

**Graphical Abstract:**

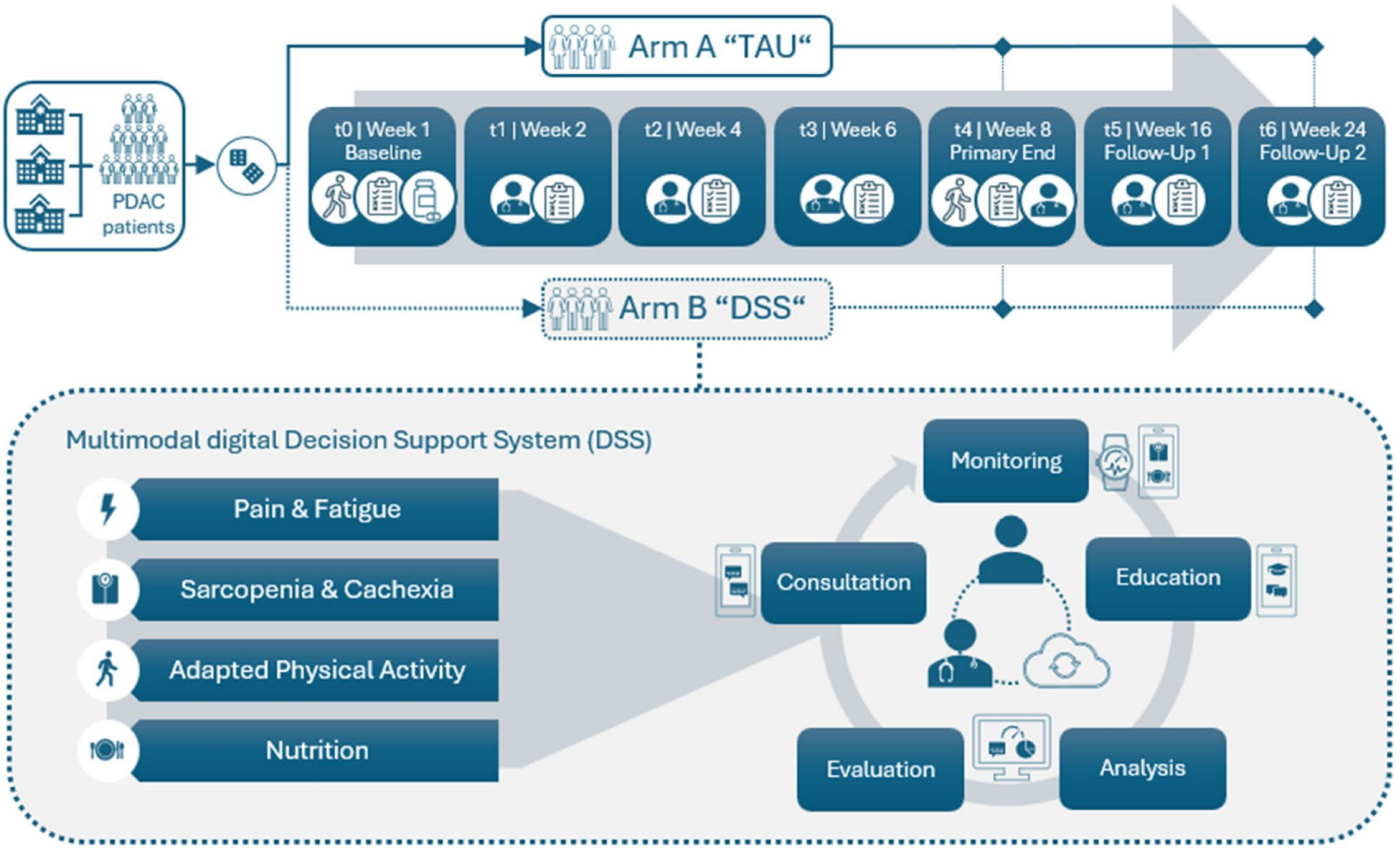

## Introduction

### Background and rationale

Pancreatic cancer is one of the most lethal malignancies, characterized by late-stage diagnosis and limited treatment options, necessitating comprehensive interdisciplinary patient care [1, 2]. Across Europe, pancreatic cancer shows an increasing incidence, including in patients under 50 (early onset cancers) [3]. Patients affected by pancreatic cancer are in high need of low-threshold, accessible, supportive and palliative care in combination with anticancer therapy early in the treatment process, aiming to improve their health-related quality of life (HRQoL) [4, 5]. Integrating multimodal supportive care, including nutritional support, physical exercise prescription, and pain management alongside digital health methods, offers a promising approach to addressing this complex therapeutic challenge [6–8].

Pancreatic ductal adenocarcinoma (PDAC) is the most common histological type (90% of cases). Most patients are diagnosed at an advanced stage where the disease has progressed beyond the initial localized tumor and has spread to nearby tissues (making it unresectable) or metastasized to distant organs [9]. The prognosis for PDAC is generally poor [10]. Only a Minority of patients is eligible for surgery, which is the only potentially curative therapeutic option, and the 5-year survival rate for all stages taken together is around 10% [11]. Limited contributing factors to the low survival rate encompass the high prevalence of late-stage diagnoses, occurring in over 80% of cases [9].

Symptoms related to PDAC can be epigastric or back pain, nausea, vomiting, bloating, abdominal fullness or change in stool consistency and/or gastric outlet or bowel obstruction, weight loss, anorexia, psychological and social distress, depression, new-onset diabetes, or venous thrombosis and/or pancreatic enzyme insufficiency, malabsorption of fat, and occasionally pancreatitis [12, 13]. Malnutrition, as a consequence of the imbalance between energy and protein uptake and expenditures, is highly prevalent in PDAC patients (60%−70%). Malnutrition is often multifactorial, due to tumor-related symptoms and treatment toxicities. In addition, sarcopenia (i.e. reduction in muscle mass and/or function, due to malnutrition and reduced physical activity) is a critical concern in PDAC patients as it contributes to diminished treatment tolerance, impaired recovery, and worse HRQoL and overall survival [14]. Moreover, cachexia (i.e. a complex syndrome of loss of muscle and fat mass driven by chronic inflammation) is a common issue of PDAC patients and is associated with reduced performance status, impaired mobility, higher morbidity, and reduced survival [15, 16]. Pain is also very common in PDAC patients and negatively affects HRQoL; it can also impact nutritional intake and physical activity, thereby contributing to malnutrition/sarcopenia.

Therefore, interventions targeting muscle preservation, such as nutritional support and adapted physical activity (aerobic and resistance exercise), are vital to improving outcomes by countering muscle loss and enhancing treatment response in PDAC patients [14]. Nutritional support can at least slow down or partially reverse complications associated with cachexia and malnutrition if started early in the patient care pathway [17]. Thus, it is imperative to employ personalized nutritional interventions (oral, and if insufficient enteral or parenteral) in order to maintain adequate nutritional status, thereby enhance treatment tolerance, support recovery, and ultimately improve HRQoL and survival [18]. Similarly, physical exercise preserves or increases muscle mass and strength, aerobic capacity, and HRQoL, while attenuating pain and anxiety, and stimulating appetite in cancer patients [19, 20]. Ample evidence suggests that physical activity is an effective intervention to alleviate common disease and treatment complications, such as impaired physical function, reduced HRQoL, fatigue, and malnutrition [21–24].

In literature, multimodal interventions aimed at nutrition, physical activity, and customized pain management strategies seem to be effective in improving the HRQoL of patients with different cancers, such as breast cancer and advanced lung cancer [25]. However, such strategies have not been sufficiently studied for patients with advanced PDAC, who often face more complex challenges such as pain, anxiety/depression and sarcopenia/cachexia. Finally, multidisciplinary supportive care for patients with PDAC, including nutritional support, physical exercise, psychological counselling, pain management, and palliative care to improve physical, social, and psychological well-being, is essential [26].

Multiple barriers – such as the costs of interventions, frequent hospital visits, limited access to extensive cancer-center care facilities and health-professionals, and insufficient information for general practitioners and clinicians to tailor supportive and palliative care to the individual needs of patients with advanced PDAC – constrain the treatment observance and reduce patients’ HRQoL [27]. The literature on pancreatic cancer also cites various barriers to participation in diet and exercise interventions among pancreatic cancer survivors, including treatment-related fatigue, comorbidities, emotional distress, transportation difficulties, technological unfamiliarity, competing personal responsibilities, and insufficient awareness or understanding of available programs [24]. Flexible and accessible home-based or technology-supported options should be offered to overcome these barriers [24]. Studies should mainly focus on developing targeted and quickly reactive interventions to improve pain management in these high-risk groups, addressing barriers to optimal treatment, and exploring the impact of specialized care, such as that provided by gastroenterologists, on pain outcomes in PDAC patients [28].

The efficacy of multimodal care in pancreatic cancer treatment can be improved by telemedicine in integrating various aspects of patient care, including remote symptom monitoring, psychological support, patient education, psychological counselling, and postoperative follow-up [29, 30]. Furthermore, telemedicine can improve access to care, especially for patients in rural areas and there has been growing evidence for the palliative care setting that telehealth improves communication, symptom management, and patient care perspective [30, 31]. However, further research needs to assess telehealth impact on symptom relief and overall HRQoL [31]. In general, digital health is assuming an increasingly significant role in the management of non-communicable internal diseases due to innovative approaches, such as the improvement of clinical decision-making through longitudinal monitoring of health-related parameters, also for cancer [32].

Digital solutions focusing on improving the HRQoL for cancer patients, particularly through remote monitoring and empowerment, have enhanced psychosocial well-being and are well received by both patients and caregivers, suggesting their potential for routine integration in cancer care [33]. Moreover, it was recently emphasized that future digital health studies must focus on evaluating the effectiveness and implementation of digital therapeutics, addressing barriers to adoption and developing strategies to enhance interoperability and integration with existing healthcare systems [34]. Artificial intelligence has been identified as an important component in the future of telemedicine to facilitate remote monitoring systems and enhance clinical decision-making. However, artificial intelligence approaches need rigorous evaluation and standardized data frameworks to ensure safety and efficacy [35].

The European-funded project RELEVIUM aims to overcome the aforementioned barriers to effective multimodal supportive therapy in PDAC through a versatile digital support system designed to reduce sarcopenia, improve HRQoL and management of chronic and neuropathic pain, and maintain physical function. The RELEVIUM randomized controlled trial (RELEVIUM-RCT) investigates whether a personalized, digitally assisted multimodal supportive care intervention can improve the HRQoL and empower patients with advanced PDAC during chemotherapy. Additionally, it examines whether the RELEVIUM digital health system enables clinicians to tailor home-based, fast reactive, clinical guidance in optimizing the treatment of cachexia, pain, and adverse symptoms.

## Objectives

The *primary objective* of the RELEVIUM-RCT is to assess the efficacy of the respective intervention during chemotherapy on the HRQoL of patients with PDAC. *Secondary objectives* are to evaluate the potential of the proposed technology-based solutions in providing timely access to supportive and palliative care treatment interventions, to assess physical capacity, nutritional status, pain and cachexia in patients with (arm A) versus without remote monitoring and personalized guidance (arm B), to investigate the stress burden of PDAC to families and family caregivers of these patients, and to assess the cost effectiveness of the proposed multimodal intervention.

## Trial design

The RELEVIUM-RCT is designed as a controlled, randomized, multicenter trial with two parallel groups with the primary endpoint after week 8 and the secondary endpoints at follow-up week 16 and follow-up week 24.

## Methods: participants, interventions and outcomes

### Study setting

The RELEVIUM-RCT will be conducted in the following three clinical centers: the University Medical Center Mainz Department of Medicine 1 (Mainz, Germany); the Health Corporation RAMBAM, Division of Oncology (Haifa, Israel); North Estonia Medical Center, Oncology and Hematology Clinic, Chemotherapy Center (Tallinn, Estonia).

### Eligibility criteria

The RELEVIUM-RCT study population includes newly diagnosed patients with histologically confirmed advanced, unresectable, or metastatic PDAC (*n* = 132) before starting first-line standard chemotherapy. Patients > 18 are eligible; under/over 65 years (no age limit) will be included to evaluate the role of age on adherence and efficacy of the intervention. All inclusion and exclusion criteria are listed in the following tables (Tables 1 & 2). The responsible physicians of the clinical centers included in RELEVIUM screen for eligibility criteria.

**Table 1:** Inclusion criteria of the RELEVIUM-RCT

No	Inclusion criteria
01	Patients with histologically confirmed, previously untreated, unresectable (locally advanced or metastatic) advanced pancreatic cancer ductal adenocarcinoma (PDAC)
02	Eastern Cooperative Oncology Group (ECOG) performance status (PS): ≤ 2
03	Patients with measurable or evaluable lesion according to the Response Evaluation Criteria in Solid Tumors (RECIST) v1.1 criteria
04	Scheduled for combination chemotherapy (e.g., Gem-Abraxane or FOLFIRINOX)
05	Age ≥ 18 years
06	Designated physical activity partner
07	Signed and dated informed consent form
08	Valid registration in a national health care system
09	Adequate bone marrow, liver and renal function
10	Participants of other clinical studies will be eligible for the study if the primary endpoints of both studies do not overlap
11	Patient can comply with all study procedures
12	Patient willing and able to give informed consent for participation in the study
13	Patient should have a smartphone and agree to use it for the purposes of the study

**Table 2:** Exclusion criteria of the RELEVIUM-RCT

No	Exclusion criteria
01	Cardiovascular, respiratory, psychiatric, musculoskeletal, or neurological condition contraindicating physical exercise
02	Endocrine or acinar pancreatic carcinoma
03	Active cerebral metastases, a history of another active major cancer
04	Active infection
05	Chronic diarrhea
06	Clinically significant history of cardiac disease
07	Pregnancy or breast-feeding
08	Legal incapacity or limited legal capacity
09	Patients which present, in the opinion of the Investigator, a reduced cognitive function (such as dementia) or technological illiteracy and are not able to provide reliable information to the study

### Who will take informed consent?

Eligible patients need to submit their written informed consent before their enrolment into the RELEVIUM-RCT in each clinical center. The original signed informed consent will be retained at the clinical site and a copy will be provided to the patient. The patient information and the declaration of consent are translated into each local language(s) and reviewed and approved by the local ethics committee.

### Additional consent provisions for collection and use of participant data and biological specimens

Not applicable: There will not be ancillary studies.

## Interventions

### Explanation for the choice of comparators

The participants in the control group will receive standard of care. Both arms will receive combination chemotherapy (e.g., Gem-Abraxane or FOLFIRINOX) according to the investigator’s decision. To keep the control arm adherent and compliant, general health literacy, physical activity, and pain management programs will be provided.

### Multimodal study design and intervention description

#### Study design

Patients will be randomly assigned to the intervention arm A or control arm B. The multimodal approach consists of pain, nutrition, physical activity, and sarcopenia interventions. Arm A will receive a smartwatch and a mobile application to download on their smartphone, and a web application for the monitoring clinicians. The intervention arm will receive guidance by the treating physician/study nurse on physical activity, nutrition and pain management which will be adapted bi-weekly, based on the data collected through the wearable devices and the mobile app. The proposed multimodal intervention aims at providing personalized nutrition and physical activity guidance, as well as improved individual palliative care symptom management based on information obtained from wearable devices and sensors and the mobile app.

#### Pain management

The mobile patient app will be used to remotely assess participants’ pain data and to enable their longitudinal analysis on the physician dashboard. In all involved clinical centers, the pain management intervention will be executed by physicians. At baseline, all recruited patients undergo baseline assessments within 7 days before the first chemotherapy cycle. At the first clinical visit the patients receive initial counselling and advice on pain management. The complete pain medication history will be assessed and the patients answer to the pain disability index (PDI) [36].

After the baseline assessments, the patients of the control arm will be managed as usual. The intervention arm will additionally be introduced to the functions of the smartwatch and mobile App including the required actions for remote monitoring, the provided educational material and individualized counselling for pain management. The patient mobile App allows remote pain monitoring through two main functions. On a numeric rating scale (NRS [Scale 0–10]), the patients shall rate their pain every day.

The patients wear the smartwatch on their wrist throughout the day. The data measured by the smartwatch and entered in the patient mobile App will be continuously visible on the physicians’ dashboard as longitudinal pain data. Alerts will be visible if NRS is > 5 and/or certain heart rate variability (HRV) or electrodermal activity (EDA) peaks during the day. Besides the summary of the adjusted pain medication, educational material about general pain medication counselling or information is stored in the patient mobile App. Such material will be provided as links to national open-source platforms (e.g., Website, Portable Document Format, Video, Podcast).

#### Nutrition

The nutritional part of the intervention follows the European Society for Clinical Nutrition and Metabolism (ESPEN) guidelines [37, 38]. The aim is to achieve a calorie intake of 30 kcal/kg/day (± 15%) and 1.5 g (± 20%) of protein/kg/day by standard/enriched oral feeding with dietetic counselling ± Oral Nutritional Supplements (ONS) ± enteral and/or, parental nutrition. Nutritional support as usual shall be improved by the application of a digital infrastructure consisting of a patient mobile App, and a physician dashboard. The digital approach allows longitudinal remote monitoring of patients’ nutritional status, also during chemotherapy cycles. In all involved clinical centers, a dietician will support the nutritional intervention. At baseline, all recruited patients undergo baseline assessments within 7 days before the first chemotherapy cycle.

At the first clinical visit, the patients receive initial counselling and advice on nutrition. Thereby, the nutritional risk for malnutrition will be assessed based on the Global Leadership Initiative on Malnutrition (GLIM) criteria [39]: non-volitional weight loss, low body mass index, or reduced muscle mass and function (Ultrasound). In addition, biological markers (C-reactive protein, albumin, pre-albumin) will be collected and the patients together with the dietician answer Patient-Generated Global Subjective Assessment (PG-SGA) [40, 41]. After the baseline assessments, the patients of the control arm will be managed as usual.

The intervention arm will be introduced to all functions of the mobile App including the actions required for remote monitoring and the educational material and counselling provided for nutrition. At home and via the patient mobile App, the patients of the interventional arm assess the PG-SGA-Short Form (PG-SGA-SF) [42] the day before each chemotherapy cycle. Furthermore, the patients rate their nutritional intake via the visual analogue SEFI scale [43] in the patient mobile App every day of the intervention. Voluntarily, the patients can document their nutritional intake with a 1–3-day photo nutrition diary in the patient mobile App. In addition, the patients have the possibility to enter adverse side effects (e.g., nausea/vomiting, diarrhea) in the patient mobile App.

All nutrition data entered in the patient mobile App are visible in the physician dashboard at any time. In addition, the date of chemotherapy cycles, medication, and adverse side effects are visible. Baseline data and longitudinal data on body mass, PG-SGA-SF and SEFI scale will be shown. Alerts will be visible if PG-SGA-SF is < 4, if SEFI scale is < 7 for more than two days or loss of > 2% of the initial body mass occurred. If alerts occur, physicians receive an automated message to commission a dietician to repeat and adjust the initial advice on nutrition during the biweekly visit in the clinical center. A short summary of the adjusted nutritional advice will be created on the physician dashboard and sent to the patient mobile App. There will not be automated counselling for food intake via push notification on the patient mobile App. Besides the summary of the adjusted nutrition advice and counselling, educational material about general nutrition recommendations or information for PDAC patients is stored in the patient mobile App. Such material will be provided as links to national open-source platforms (e.g., Website, Portable Document Format, Video, and Podcast). At biweekly visits, C-reactive protein and albumin will be measured. After the intervention, the nutritional risk for malnutrition and the PG-SGA will be assessed again at each clinical center.

#### Physical activity & exercise

The physical activity intervention aims to improve the patients’ HRQoL due to the beneficial effects of regular, tailored physical exercise on pancreatic cancer related pathophysiology and symptoms (e.g., fatigue, sarcopenia) and to test the feasibility of the improved remote monitoring approach in all clinical centers. Within 7 days before the first chemotherapy cycle, patients from both arms of the RELEVIUM-RCT perform the physical assessments to determine their physical condition. These include: the International Physical Activity Questionnaire Short Form (IPAQ-SF) [44], the Physical Activity Readiness Questionnaire + (PAR-Q +) [45], the Six Minute Walk Test (6MWT) [46], the Five-Times-Sit-to-Stand-Test (FTSST) [47, 48], the Handgrip Strength Test (HGST) [49, 50], and the Brief-BESTest (BBT) [51, 52].

After baseline assessment, all patients receive initial advice and recommendations on physical activity. Then, the control arm is managed as usual. In contrast to usual care, the intervention arm receives a smartwatch, access to the patients’ mobile App, resistance bands, and an introduction about all the functions of the smartwatch, the mobile App, training materials and their remote physical activity program. Additionally, the patients will be encouraged to designate a physical activity partner (a healthy family member/relative or friend) who can attend (without obligation of performing exercises) according to Neuzillet et al. (2015) [53] Exercise therapist or physiotherapist or study nurse guide these baseline measures in each clinical center.

The individual initial training recommendations for the 8 weeks of intervention are based on the results of the physical assessment tests, the individual questionnaire data, and the ACSM guidelines [54]. After the baseline assessments, physical activity and exercise guidance, remote monitoring, and education will be provided through the patients’ mobile App and smartwatch. Physical activity analysis and adjustment of the counselling will be conducted via the physicians’ dashboard assisted by the digital decision support system.

In detail, the patients wear the smartwatch on their wrist throughout the day. The patients’ smartwatch measures daily physical activity, heart rate (HR [bpm]), steps, exercise type, time [min], frequency [n]. After each exercise session, the patients actively rate their perceived exertion (RPE [scale 0–10]), and exercise-related pain (RPP [scale 0–10]) via the push buttons of the smartwatch. Data of the smartwatch is continuously synchronized with the patients’ mobile App and the backend.

In the patients’ mobile App, the patients can find their individual training suggestions. In addition, the protocol of their daily physical activity and the performed training sessions is displayed in the mobile App. Thereby, the participant can prove the correctness of the protocol and if needed add a comment. In addition, comments can be added on the exercise experience or current issues related to physical activity. The recorded training diary is automatically sent to the server. From the server, the diary will be sent to the physicians’ dashboard including an automatic evaluation considering patients implementation of the training suggestions.

The physical activity data and the exercise session data appear on the dashboard. Longitudinal data on daily physical activity, frequency, intensity, type, and time of performed exercise sessions including RPE and RPP can be viewed on the dashboard at any time. Also, the result of the automatic evaluation and the new automatically derived suggestion appear on the dashboard. The physician or the healthcare professional can proof the evaluation based on single session analysis and accept or adjust and send the new suggestion to the patients’ mobile App. Alerts will appear on the dashboard if RPP > 3 or RPE > 7 or if a low adherence to the training program < 50%.

According to the current consensus regarding physical activity of patients with pancreatic cancer, the patients will mainly be suggested to perform unsupervised, home-based, moderate aerobic training sessions. The patients receive a specific suggestion how long, how intensive, and how frequent they should perform these endurance training sessions. Each week the patients are suggested to perform up to 150 min (3–5 sessions) of moderate aerobic training. The session can be conducted either as a continuous session or as 30/30 interval training. Aerobic training can be carried out in the form of two 15-min training sessions per day at low intensity to avoid bed rest in the first week after chemotherapy. All training sessions consist of personalized aerobic and resistance exercises and contain a warm-up and recovery phase of 30% of the complete training session according to Neuzillet et al. [53]. The warm-up includes ten dynamic stretching exercises (neck adduction, neck flexion/extension, neck rotation, shoulder rotation, elbow flexion, wrist rotation, hip flexion, hip adduction, knee flexion, and ankle rotation) [55]. Aerobic training ranges from walking, walking, and/or cycling to running, according to the patient's preference and physical capacity. The training duration is gradually increased to 30 min. Depending on their physical fitness, patients will perform aerobic exercises at an intensity that will allow the patient to speak comfortably (walk briskly, RPE 4–6) [53].

Additionally, exercise sessions contain strength exercise with resistance bands or their own body weight, flexibility, and coordinative exercises twice a week. According to Naito et al. [56] and Solheim et al. [57], the individual exercise program consists mainly of three to five of the following five exercise components. There are two training components (Training Session 1 and Training Session 2). Training Session 1: (1) push-ups against the wall, (2) bicep curls, (3) rowing, (4) squats, (5) calf raises. Training Session 2: (1) overhead presses, (2) butterfly reverse, (3) good mornings, (4) lunges, (5) rotation. The exercises are performed with 2–3 sets and 8–12 repetitions on each side. Based on the RPE, the resistance training will be adapted (RPE < 3 increase intensity, RPE > 6 decrease intensity). A certain amount of the exercise sessions will be delivered as training descriptions which can be accessed via the mobile App for patients. The additional exercise sessions do not last longer than 15 min and can be added to the endurance exercise twice a week or be performed instead of the second endurance bout depending on individual preference.

Before the patient starts training, the appropriate training session is selected on the smartwatch. After each training session, patients rate their perceived exertion and perceived pain. A warning appears in the physicians’ dashboard if the RPE value is greater than 7 or if the patient reports pain immediately after the exercise bout (RPP > 3). According to Neuzillet et al. [53], the physical activity intervention will be discontinued if: uncontrolled infection, thromboembolic event (first 7 days of anticoagulant therapy), uncontrolled pain, symptomatic anemia or hemoglobin level < 10 g/dl, platelet counts < 50,000/mm3, and a major surgery occur and on patient’s request, deterioration of general condition with ECOG performance status > 2, uncontrolled symptoms, clinical problem definitively contraindicating exercise, clinicians’ or sports therapists’ decision, serious adverse event related to exercise, loss to follow-up, and major protocol deviation.

Besides the adjusted training counselling, educational material about the effects of regular physical activity on pancreatic cancer and descriptions of certain exercises is stored in the patient mobile App. The participants receive information about the benefits of physical activities related to their diseases with the aim to increase the compliance and motivation to perform physical activity on a regular basis. The information will be provided in the form of a text. Such material will be provided as links to national open-source platforms (e.g., Website, Portable Document Format, Video, Podcast).

#### Sarcopenia, body composition and nutritional status

**Fig. 1:**
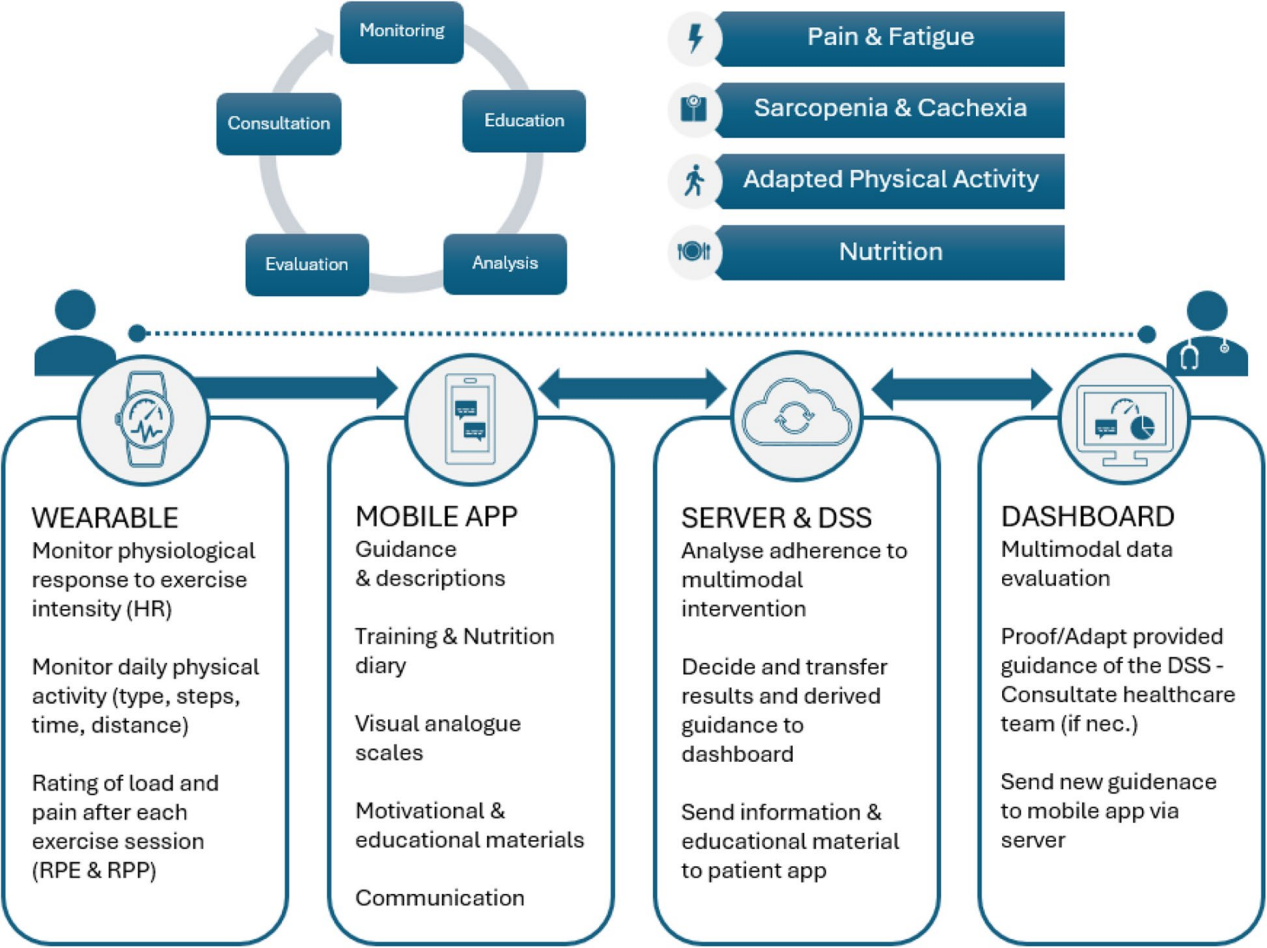
Overview of the digital-assisted supportive care intervention and monitoring

The physical activity and nutrition intervention include assessments related to sarcopenia (e.g., HGST, 6MWT, FTSST, PG-SGA). As an additional specific remote monitoring tool, a score to predict the sarcopenia risk, the SARC-F [58], will be assessed weekly through the patients’ mobile App. Alerts will be created in the physicians’ dashboard if the score is > 3. The ultrasound images, taken at visit 1, 3 and end-of-study visit, will be used to train and validate machine learning algorithms that will be able to automatically segment the muscle area and measure the muscle thickness of the muscle. The CT scans at the start and the end of the study, with the use of the third lumbar cross-sectional muscle evaluation will provide the ground truth to evaluate the accuracy of the algorithms. The data concerning age, sex, BMI, HGST, 6MWT, FTSST will also be used in the training of the algorithms in order to enhance the prediction accuracy in a multimodal manner. Output of the algorithm will be the muscle thickness (MT) and the estimation of the presence of sarcopenia, which will be visible in the physician’s dashboard. An overview of the components and functions of the digital-assisted supportive care system is shown in Fig. 1.

### Criteria for discontinuing or modifying allocated interventions

The study will not be continued if the patient requests to discontinue the study, or if the disease progression is so severe that the patient is incapable to use the RELEVIUM system, or the patient's state of health requires an ECOG classification that strongly deviates from the inclusion criteria.

### Strategies to improve adherence to interventions

Close monitoring and regular targeted clinical advice for patients should increase adherence. Above all, the personal exchange with the physicians and healthcare team during the biweekly clinical visits and the education on the effects of the supportive therapy via the mobile App should encourage the patients to follow the intervention. A partner from the patient's social environment is also involved at the beginning of the study to accompany the recommended physical activities during the study.

### Relevant concomitant care permitted or prohibited during the trial

N/A: All patients should follow the usual treatment regimen. Monitoring allows patients to pursue all activities of their daily life and enables consideration in final data analysis and interpretation.

### Provisions for post-trial care

Upon the termination of the study or discontinuation of participation attributable to declining performance status or other considerations, patients will subsequently receive ongoing care at the respective clinical center, in accordance with standard clinical practices.

### Outcomes

#### Study Outcomes

**Fig. 2:**
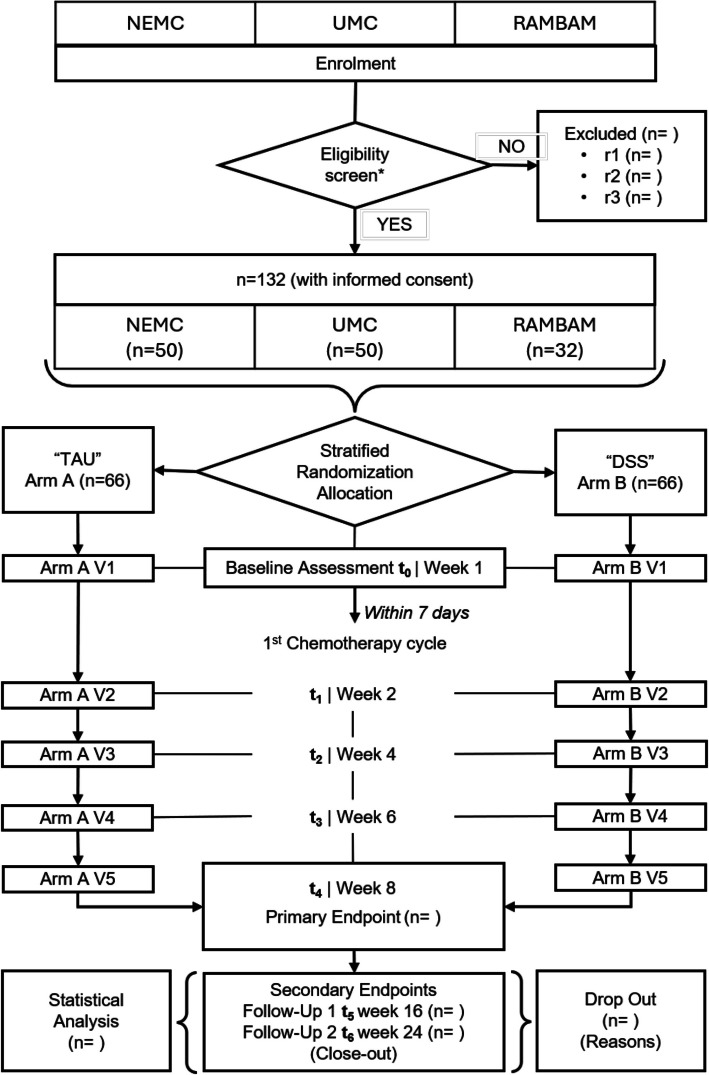
Overview of the participant timeline. *Legend*: UMC: University Medical Center Mainz, GER, NEMC: North Estonia Medical center, Tallinn, Estonia; RAMBAM: Rambam Healthcare Campus, Haifa, Israel; TAU: Treatment as usual; DSS: Multimodal digital Decision Support System: *: s. eligibility criteria {10}

After determining eligibility to participate in the study, patients are randomly assigned to the two study groups and start with the baseline tests. The first chemotherapy cycle then begins. Patients visit the clinical center every two weeks. The primary endpoint is after week 8 of intervention and the secondary endpoints after a follow-up at week 16 and week 24 after the start of the study. All outcomes are listed in Table 3 and the participant timeline is shown in Fig. 2

**Table 3:** Overview of all RELEVIUM-RCT outcomes, parameters, instruments, and assessments

Outcome	Parameter	Instrument	T0	T1	T2	T3	T4	F-U1	F-U2
**Primary Outcome**									
Health-related QoL	Score [0–100]	EORTC-QLQ-C30	x	x	x	x	x	x	x
**Secondary Outcomes**									
Patients-reported outcomes									
Psychosocial Determinants	Likert Scale [1-5]	SIIQ	x		x		x		
Financial determinants	Likert Scale [1-5] close-ended questions	SIIQ							
Patient & Caregiver Acceptance	Score [0–84]	BSFC-S	x		x		x		x
Nutrition	Score [0–12]	PG-SGA	x				x		
Physical activity	MET [min/week]	IPAQ-SF	x				x	x	x
Pain	Index [≥ 33]	PDI	x	x	x	x	x		
Sarcopenia	Score [0–10]	SARC-F	x						x
Physical measurements									
Anthropometry	[kg], [m]	Body mass, Height	x	x	x	x	x	x	x
Vital signs	[mmHg], [bpm], [bpm], [°C]	Blood pressure, heart rate, respiratory rate, temperature	x	x	x	x	x	x	x
Laboratory parameters	[mg/dl], [mg/L]	C-Reactive Protein, Albumin and Pre-albumin	x	x	x	x	x	x	x
Tumor Type and Staging	CT scan [mm]	RECIST Criteria	x				x		x
Muscle thickness	[cm]	Ultrasound Images	x		x		x		x
Physical function	Walking distance [m]	6MWT	x				x	x	x
	Handgrip-strength [kg]	HGST	x				x		
	Time [sec]	FTSST	x				x		
	Score [0—24]	BBT (overall)	x				x		
	Biomechanical constraints [Score 0–3]	BBT (Hip strength)	x				x		
	Stability limits/verticality [Score 0–3]	BBT (Reach forward)	x				x		
	Anticipatory postural responses right [Score 0–3]	BBT (one limb stand right)	x				x		
	Anticipatory postural responses left [Score 0–3]	BBT (one limb stand left)	x				x		
	Postural responses right [Score 0–3]	BBT (Stepping right)	x				x		
	Postural responses left [Score 0–3]	BBT (Stepping left)	x				x		
	Sensory orientation [Score 0–3]	BBT (Stance on foam with eyes closed)	x				x		
	Stability in gait [Score 0–3]	BBT (Get up and Go test)	x				x		
Efficacy									
Adherence patients	[n] & [min], [n], [min], [RPE]	App logins & time, Smartwatch data (Exercise sessions, duration and load)							
Adherence clinicians	[n] & [min]	Dashboard logins & time, messages send out							
Usability	Likert Scale [1–5] close-ended questions	Usability Questionnaire in SIIQ							
Cost-Effectiveness	Cost-Effectiveness Ratio	Cost Effectiveness Questionnaire in SIIQ							
**Additional-Daily remote measurements via mApp:** VAS Fatigue, VAS Pain, VAS Nutrition									
**Additional-Weekly remote measurements via mAPP:** SARC-F, PG-SGA-SF									

7 days prior 1 st chemo cycle, Week 2 ± 5 days, Week4 ± 5 days, Week6 ± 5 days, Week8 ± 5 days, Week 16 ± 1 week, Week 24 ± 1 week, SIIQ: Social impact index, BSFC-S: Burden Scale for family caregivers, 6MWT: Six-Minute Walk Test, HGST: Handgrip Strength Test, FTSST: Five Times Sit to Stand Test, BBT: Brief-BESTest, VAS: visual analogue scale, SARC-F: Strength Assistance (with Walking) Rising (from a Chair) Climbing (Stairs) Falls, PG-SGA: Patient-Generated Subjective Global Assessment

*PDI *Pain Disability Index, *IPAQ-SF *International Physical Activity Questionnaire—Short Form, *EORTC-QLQ-C30 *European Organization for Research and Treatment of Cancer Quality of Life Questionnaire

### Participant timeline

#### Sample size

Acknowledging the potential challenges posed by the severity of illness, a dropout rate of 20% is assumed. Consequently, the calculations were done by the R package power.mmrm with an equal dropout pattern up to 20% in each treatment arm as correlation structure compound symmetry with a correlation of 0.5 between measurements was used. The significance level was set to α = 5% and the power to 80%. With 66 patients in each arm a standardized effect size of about d = 0.52 can be shown. When assuming a standard deviation of 23.6 score points mean of the standard deviations of women and men this amounts to a difference between arms of 12.3 score points which can be considered as clinically relevant [59]. Conservatively, the correlation between the EORTC-QLQ-C30 and the stratification factors ECOG and age were assumed to be 0 for planning since there is no reliable correlation estimation available.

### Recruitment

In all clinical sites, recruiting staff members are informed in detail about all randomized controlled trial measures and trained in an effective recruitment strategy.

## Assignment of interventions: allocation

### Sequence generation, Concealment mechanism, Implementation

Centralized Randomization will be performed applying the secure Research Electronic Data Capture (REDCap) randomization module [60]. Thereby, the allocation sequence will be generated and stored within the system. The responsible investigator accesses the system to automatically and randomly assign a participant without revealing the sequence. Thus, the investigator cannot influence or predict the next assignment. The allocation sequence will be generated by computer-based random number generation using R statistics. Randomization will be stratified by ECOG performance status (0, 1, 2) and age (≤ 65 vs. > 65). All details of sequence generation are documented separately in a restricted-access randomization specification file. This document is unavailable to individuals involved in participant enrolment or group assignment to ensure concealment and minimize bias.

## Assignment of interventions: blinding

### Who will be blinded

No blinding of the individuals in the healthcare team and the patients is possible due to the nature of the intervention. Data analysis is performed under blinded conditions, based on anonymized data.

### Procedure for unblinding if needed

N/A (s.).

## Data collection and management

### Plans for assessment and collection of outcomes

#### Primary outcome

The primary outcome of the RELEVIUM-RCT is HRQoL assessed by the EORTC-QLQ-C30 including longitudinal analysis such as time to definitive deterioration of one of the selected dimensions physical functioning or/and appetite loss. The European Organization for Research and Treatment of Cancer-Quality of Life-C30 questionnaire (EORTC-QLQ-30) is a valid, 30 items questionnaire to assess HRQoL of cancer patients [61]. It covers five functional scales (physical, role, cognitive, emotional and social), nine symptom scales (fatigue, pain, nausea and vomiting, dyspnea, insomnia, appetite loss, constipation, diarrhea, financial difficulties) and global health and quality of life scale (version 3.0). The scale ranges from 0 to 100, with a higher scale value representing a higher response level [62].

#### Secondary outcomes

The secondary outcomes include measures related to pain, nutrition, physical activity and physical function, fatigue, sarcopenia, sociodemographic factors, and the burden of the family caregiver.

Pain will be assessed with the PDI [7]. The PDI is a reliable and valid measure of pain-related disability and contains 7 items to measure aspects of patients’ life disrupted by chronic pain (Family/Home Responsibilities, Recreation, Social Activity, Occupation, Sexual Behavior, Self-Care, and Life-Support Activities) assessed via rating scale (0–10). Additionally, patients are asked to rate their pain on a NRS scale of 0 to 10, with 0 being "no pain" and 10 being "worst possible pain", on the mobile App [36]. NRS is a clinically accepted instrument to measure pain [63].

Nutrition will be assessed with the PG-SGA. PG-SGA includes the assessment of body mass, intake, symptoms, functional status, disease state, metabolic stress in oncology and other chronic catabolic conditions [42]. The PG-SGA consists of two parts. A specialist (doctor, nurse or nutritionist) completes the worksheets of the questionnaire. PG-SGA-SF, which consists of scores assigned to questions divided over four boxes, addressing body mass (Box 1), food intake (Box 2), symptoms (Box 3), and activities and function (Box 4), is completed by the patient [41]. Based on the numeric scores from the four boxes, patient risk for malnutrition is categorized as low (0–3 points), medium or high (≥ 4 points), or high (≥ 9 points) [42]. In addition, food intake is measured using the SEFI (Simple Evaluation of Food Intake) scale on the mobile App, which provides a visual estimate of food intake using a visual analogue scale from 1 to 10 and a visual assessment of consumed portions [64]. The SEFI scale is self-reported. Less than half of the meal or a value of less than 7 indicates malnutrition or the risk of malnutrition [43]. Moreover, a Pancreatic Enzyme Replacement Therapy (PERT) check will be carried out monitoring enzyme replacement intake by directly asking the patients [65, 66].

Physical activity will be assessed with the IPAQ-SF. The IPAQ-SF has acceptable validity and reliability compared to other self-reported physical activity measures [44]. The questionnaire contains 6 questions on moderate and vigorous physical activity and one question on daily sitting time for the last 7 days. The PAR-Q + will be applied to determine whether participants have barriers or contraindications to participate in physical activity, including heart disease, chest pain during activity or at rest, balance problems or dizziness. The PAR-Q + consists of 7 questions to be answered with "yes" or "no" [45]. Additionally physical function will be assessed by several physical tests.

The 6MWT will be performed according to the guidelines of the American Thoracic Society including the standardized instructions and is a reliable and valid submaximal test for cancer patients to assess functional capacity [46, 67]. Within six Minutes, the participants walk at self-paced speed to achieve their maximum distance. The patients can rest and adapt their speed whenever they like during the test. The primary outcome of the test is the maximum distance achieved within 6 min [m]. The materials needed are a walking course (30 m, flat, indoor, marked every 3 m), a countdown timer, two small cones to mark the turnaround points, Borg scale [0–10], Blood pressure monitor, heart rate monitor, lap counter, and colored tape.

The FTSST will be performed to validly assess the dynamic balance and the functional mobility [48]. The outcome of the FTSST is associated with frailty in cancer patients, is valid and reliable to measure lower body function [47]. As a start position, the participants sit on a chair with their arms folded across their chest. With the start signal the participants rise from the chair and return to the seated position as quickly as possible for five repetitions. The test will be repeated after a short break of one minute and the mean time of both repetitions will be used for the analysis. The chair should not be placed against a wall. The outcomes of the test are the time needed to complete five repetitions (Time [sec.]) and issues that occurred during the test or (e.g.: assistance was needed, not able to complete five rep.). The materials needed are an armless back chair (16 inches), stopwatch, tablet.

The HGST will be performed. Handgrip strength (HGS) is associated with overall cancer mortality in both males and females [49]. Also, in combination with the assessment of muscle mass, HGS is an indicator of sarcopenia [68] and HRQoL [50]. The HGS will be measured using a dynamometer (Jamar). The handles of the tool have to be pressed together once to the maximum with the dominant arm. The participants will perform the test twice, with a rest of three minutes between the two repetitions. The mean value of two trials is recorded. The primary outcome is the maximum force produced. The materials needed are Jamar dynamometer, chair, stopwatch, tablet.

The BBT is a clinical balance assessment tool. It is a 6-item revised, abbreviated version of BESTest [51, 69] designed to assess six different aspects contributing to postural control in standing and walking. The BBT is quick and easy to perform [52]. Each item is scored from 0–3 points (0 = severe impairment, 3 = no balance impairment). The total score of the BBT is 24 points (2 items include both R/L components). Six tests are included in the BBT (8 scored): Biomechanical constraints: Hip strength, Stability limits/verticality: Reach forward, Anticipatory postural responses: Stand on one limb: left and right each scored, Postural responses: Compensatory Stepping right and left each scored, Sensory orientation: Stance on foam with eyes closed, Stability in gait: Timed Up & Go test. The materials needed are stopwatch, meter stick, space to complete the Timed Up & Go Test, stable chair, Medium density 4-inch foam pad (e.g., Airex).

Sarcopenia will be assessed with the SARC-F, which is a short 5-item internally consistent and valid questionnaire [58] recommended by the EWGSOP2 guideline [70] on definition and diagnosis of sarcopenia. Cancer patients undergoing chemotherapy often experience fatigue because of chemotherapy-induced anemia. The fatigue visual analogue scale provides a simple method to assess fatigue daily via the mobile App [71]. Patients will also wear the smartwatch for longitudinal monitoring of health-related parameters (e.g., HRV & EDA).

For social impact, a social impact survey was developed by the RELEVIUM project members to identify and address significant social and economic barriers. Additionally, the BSFC-S [72] will be applied to assess the subjective burden of informal caregivers.

Considering patient condition, at baseline and after the intervention the following scales should be administered by following order: 1. EORTC QLQ-C30; 2. PDI; 3. PG-SGA; 4. Social Impact Index Questionnaire; 5. IPAQ-Short Form; 6.PAR-Q +; 7. SARC-F.

#### Plans to promote participant retention and complete follow-up

Patient engagement strategies will be applied to reduce participant dropout rates, utilizing patient-friendly materials and regular communication channels to keep participants informed and motivated.

#### Data management

For registering clinical data, an electronic case report form (eCRF) system will be applied via REDCap, a secure electronic data capture (EDC) system. The estimated dataset size for the RCT is 1TByte, but it significantly depends on patient compliance. Study investigators/sites will enter data directly into an EDC system by completing the eCRF via a secure internet connection. If possible, data entered into REDCap must be verifiable against source documents at the study site. Any changes to the data entered in REDCap will be recorded in the audit trail. Data will be evaluated for compliance with the protocol and accuracy in relation to source documents. Authorized clinical research associates will verify that the trial is conducted, and data are generated, documented and reported in compliance with the protocol, good clinical practice (GCP) and the applicable ethics and regulatory requirements. Data coding procedures are executed and captured via REDCap to ensure consistency, standardization, and ease of analysis. Branching logic and calculated fields within REDCap are utilized to automate data coding wherever possible, minimizing errors and ensuring consistency. Data entry fields in REDCap are configured with validation rules (e.g., numeric ranges, dropdowns with predefined options) to enforce correct coding. All collected data including sensor readings and questionnaire responses, are securely transmitted to a cloud-based platform. This ensures data integrity and confidentiality while allowing for scalable storage and access. Secure storage also facilitates data retrieval and analysis by healthcare providers, ensuring that patient information is always up-to-date and easily accessible. All Investigator/sites must maintain primary source data of all study related documentation, including medical records, examination reports and other relevant medical reports in written or in electronic form. Processes to promote data quality (e.g., double data entry, range checks for data values): The data monitoring committee (DMC) will independently assess data quality. Real-time monitoring is implemented to identify and rectify data discrepancies promptly. A centralized data monitoring system is employed for real-time tracking with regular review and reconciliation of data at predefined intervals. Ongoing training sessions for study personnel on the importance of complete data collection will be conducted, and a communication protocol will be established to promptly address data collection issues. Data recorded at the clinical sites will be verified against the initial data records by the contract research organization (CRO). A Data Management Plan (DMP) is utilized to ensure the systematic handling of data. All data collected during the study will be managed using REDCap. Data handling and processing will comply with the General Data Protection Regulation (GDPR) to safeguard the privacy and integrity of participant information.

#### Confidentiality

The study will comply with the GDPR and Data Protection Act 2018. The processing of the participants’ personal data will be minimized. All documents will be stored securely and only accessible by study staff and authorized personnel. The study staff safeguard the privacy of participants’ personal data. Participants will be assigned a unique code identifier by the system. Any records or datasets that are transferred to the study database will contain the code identifier only; participant names or any information, which would make the participant identifiable, will only be held locally and securely at the respective clinical site and will not be transferred.

#### Plans for collection, laboratory evaluation and storage of biological specimens for genetic or molecular analysis in this trial/future use

N/A: Biological specimens will not be used in this study.

## Statistical methods

### Statistical methods for primary and secondary outcomes

The primary efficacy objective is to evaluate the AI-guided multimodal intervention, measured by EORTC-QLQ-C30 and assess the improvement of HRQoL within period of 24 Weeks, between Baseline Visit (Week 1) and the Follow up Visit (Week 24) in adult patients with Pancreatic Cancer.

The primary estimate linked to the primary objective is defined by the following 4 attributes:

1. Population of interest: full analysis set; all randomized patients; intention-to-treat policy as randomized. The patient population is defined by the inclusion/exclusion criteria.
2. Variable of interest: HRQoL assessed by change in the EORTC-QLQ-C30 (including longitudinal analysis such as time to definitive deterioration of one of the selected dimensions physical functioning or/and appetite loss) from baseline to end of Follow-up.
3. Treatment: Multimodal intervention patient arm A or Controls arm B, of the ECOG status 0, 1 and 2 and the age group below 65 years or the age group above 65 years).
4. Population-level summary statistic: Hazard Ratio for HRQoL between the two groups (as randomized to Multi-modal intervention patient arm A or Controls arm B).

#### Statistical methods for primary objectives

The primary population for the analyses of efficacy is the intention-to-treat population. The hypothesis will be tested on a two-sided level of significance α = 0.05. The primary endpoint is the change in the EORTC-QLQ-C30 from baseline to end of Follow-up. The hypotheses to be tested are: H_0_: µ_A_ = µ_B_ vs. H_1_: µ_A_ ≠ µ_B_, where µ_A_ and µ_B_ are the expected values of the change in EORTC-QLQ-C30 from baseline to end of Follow-up of arm A and arm B, respectively (superiority hypothesis). The primary endpoint will be analyzed with a generalized mixed-effect model including ECOG status (0, 1 or 2) and age (≤ 65 vs. > 65), arm, visit, and the interaction term of arm and visit as fixed effects, as well as baseline value as continuous covariate. Compound symmetry will be assumed. Model assumptions will be checked by residual plots. The test used is a linear contrast between the arms after the end of Follow-up and uses a t-statistic. Arm differences will be displayed by adjusted mean differences and 95% confidence intervals. Descriptive statistics will also be provided over time by arm and in total. The primary parameter will also be displayed over time by a boxplot. Sensitivity analyses will be done by adding additional variables like gender to the analysis model.

### Statistical methods for secondary objectives

#### Evaluation of technology-based solutions for supportive and palliative care

The evaluation of the potential of technology-based solutions in providing timely access to supportive and palliative care treatment interventions will employ a mixed-methods approach. Quantitative data on the frequency and timeliness of access to interventions will be analyzed descriptively. Additionally, qualitative data, gathered through participant interviews or surveys, will be thematically analyzed to provide in-depth insights into the participants' experiences and perceptions of the technology-based solutions.

#### Comparative analysis of pain and cachexia evolution

A comparative analysis of the evolution of reported pain and cachexia items between patients in Arm A (with remote-monitoring and personalized guidance) and Arm B (without these interventions) will be conducted using appropriate statistical tests. A longitudinal analysis employing linear mixed-effects models will be utilized to assess the trajectory of pain and cachexia over time. This analysis will consider fixed effects such as treatment arm, time, interaction of arm and time, and relevant covariates.

#### Assessment of costs and cost-effectiveness

The assessment of costs and cost effectiveness of the proposed detailed monitoring solutions will involve a comprehensive economic analysis. Direct costs associated with the implementation and maintenance of monitoring solutions will be collected and analyzed. Cost-effectiveness ratios will be calculated, comparing the costs incurred with the results achieved. Sensitivity analyses will be performed to assess the robustness of economic evaluations.

#### Chronic stress burden on families and caregivers

The assessment of the chronic stress burden on families and family caregivers of PDAC patients will employ validated psychological assessment tools. Descriptive statistics will summarize stress levels, and comparative analyses will explore potential differences between different caregiver groups. Qualitative data from interviews or surveys will be thematically analyzed to provide a nuanced understanding of the chronic stress experienced by families and caregivers. These statistical methods align with the study's protocol and aim to rigorously address the specified secondary objectives, ensuring a comprehensive and scientifically sound analysis of the proposed technology-based solutions, pain and cachexia evolution, costs, and chronic stress burden.

### Interim analyses

An interim analysis, prior to the analysis of the primary endpoint after 2 months, is not planned.

### Methods for additional analyses (e.g. subgroup analyses)

#### Subgroup analysis

Subgroups will be defined in the statistical analysis plan based on the stratification factors. Subgroup analyses will be performed by mixed models similar to the primary analysis model with additional covariates. Subgroups will be investigated by analyzing the interaction term of the subgroup and arm.

### Methods in analysis to handle protocol non-adherence and any statistical methods to handle missing data

#### Avoiding missing data

The strategy for avoiding missing data in the clinical trial involves a comprehensive and proactive approach to ensure data integrity and reliability. Key components of the strategy will include investigator training for accurate data collection and the use of EDC systems. Real-time monitoring is implemented to identify and rectify data discrepancies promptly, and a centralized data monitoring system is employed for real-time tracking with regular review and reconciliation of data at predefined intervals. Ongoing training sessions for study personnel will be conducted on the importance of complete data collection, and a communication protocol will be established to promptly address data collection issues. Thorough documentation of the reasons for missing data in eCRF will be ensured, with the use of standardized codes for reasons such as participant withdrawal or technical issues. Patient engagement strategies will be improved to reduce participant dropout rates, using patient-friendly materials and communication channels to keep participants informed and motivated. The data monitoring committee will independently assess the quality of the data and missing data patterns. A continuous improvement process will be established to review and refine missing data handling strategies based on ongoing feedback and emerging challenges, incorporating lessons learnt from previous trials or similar studies. This comprehensive and proactive strategy aims to address missing data issues throughout the clinical trial, ensuring a robust and flexible study design while maintaining data integrity and reliability.

#### Handling of missing data

Using a maximum likelihood approach can make use of cases having partially observed data without performing imputations. Mixed models can deal with missing values (at least under missing-at-random assumptions) by using the correlation structure of the observed data. Subgroup analyses are conducted to assess the impact of missing data in different participant subgroups, assessing whether missing data patterns vary by demographic or clinical characteristics.

#### Plans to give access to the full protocol, participant level-data and statistical code

Data will be published. Data will include trial procedures and anonymized data from individual patients to the extent that this is allowed by legal and ethical restrictions. Data to be considered for public access will include data from wearables, questionnaires, and possibly some important non-identifying patient information. Publications will be sent to open-access journals and editors. In all cases, the exact types and forms of data that will become openly available will be the maximum allowed to avoid violating any legal or ethical restrictions following the RELEVIUM DMP. The overall aim of allowing open access to data is to contribute to future research on effective interventions and care plans to improve the HRQoL of cancer patients in the supportive, palliative, or end-of-life setting. The statistical code will also be publicly available.

## Oversight and monitoring

### Composition of the coordinating center and trial steering committee

All relevant committees and their members are listed in the following Table 4.

**Table 4:** Composition, roles and responsibilities of all institutions supporting the RELEVIUM-RCT

Role & Responsibility	Institution	Meetings
Coordinating center & principal investigator	UMC Mainz	Weekly
Trial Steering committee*(TSC)	RAMABAM	Weekly
	NEMC	
	UMC Mainz	
Technical Management	EXUS	Weekly
Contract Research Organization (CRO)	ROSENBAUM	Weekly
Dissemination Management	FPI	Monthly
Quality Management	UMC Mainz, ROSENBAUM	Weekly
Ethics Manager & Legal Advisor	Diadikasia & external ethical advisor	
Ethics Review Committee** (ERC)	State Medical Association Rhineland-Palatinate Ethics Committee, Germany; Helsinki Committee, Rambam Health Care Campus, Israel; Ethics Committee for Human Research of the Estonian Institute for Health, Estonia	
Data Manager	ROSENBAUM, EXUS, HUA	Monthly
Data Monitoring Committee (DMC)	ROSENBAUM, Diadikasia	Monthly

^*^Each institution has lead investigator, who is also national coordinator

^**^The ERC ensures that data-gathering procedures are done based on consent forms that follow EU Regulation 2016/679 of the European Parliament and of the Council of 27 April 2016 (GDPR) on the protection of individuals concerning the processing of personal data and on the free movement of such data

### Composition of the data monitoring committee, its role and reporting structure

#### Data monitoring committee

The DMC is independent from the sponsor and has no competing interest. The CRO will implement and maintain quality assurance and quality control systems with written standard operating procedures in a clinical management plan and applicable forms/logs will be implemented at each clinical trial site, to ensure that all clinical data are generated and recorded in accordance with the protocol and in light of the Declaration of Helsinki, the International council for harmonization of technical requirements for pharmaceuticals for human use, and the guideline for GCP. Data monitoring activities will include setting up a relevant REDCap electronic data capture database and data transfer mechanisms, along with appropriate validation of data and resolution of queries.

#### Adverse event reporting and harms

Information on adverse events or serious adverse events reporting of worsening of current condition and/or laboratory results out of reference ranges as a direct result of the multimodal RELEVIUM-RCT as determined by the attending clinician will be recorded in the eCRF.

#### Frequency and plans for auditing trial conduct

The sponsor and the DMC will conduct quality assurance audits to ensure adherence to the Declaration of Helsinki, the principles of GCP, and compliance with applicable further regulatory and ethical standards. The CRO will execute site initiation, interim monitoring, and close-out visits either directly on site or through remote means. The audit strategy for trial conduct encompasses investigator training to ensure precise data collection and the employment of an EDC system. Real-time monitoring is instituted to promptly identify and address data discrepancies, while a centralized data monitoring system is utilized for real-time tracking, accompanied by regular review and data reconciliation at predefined intervals.

#### Plans for communicating important protocol amendments to relevant parties (e.g. trial participants, ethical committees)

Any deviations from the study protocol are required to be reported to the Sponsor/CRO and will be documented as an electronic record within the data monitoring system via REDCap. Intended modifications will be incorporated into all pertinent documents and registries.

## Dissemination plans

The findings of this research will be disseminated publicly through scholarly publications in open-access journals, presentations at conferences, and additional dissemination channels.

## Discussion

Our European project RELEVIUM with its newly established randomized controlled trial examines a personalized, digitally facilitated multimodal supportive care intervention for PDAC patients in three European cancer centers. The primary objective is to evaluate the effectiveness of the intervention on patient HRQoL. Secondary objectives include evaluating the capacity of technology to improve physical function, nutritional status, pain, and sarcopenia with remote monitoring and personalized guidance. Furthermore, the study aims to explore the impacts of stress on families and caregivers and to determine the cost-effectiveness of the intervention.

The study is designed to explore efficacy, usability, and cost-effectiveness of the multimodal digital decision support system. Analysis of longitudinal data will facilitate an exploration of the usability of the digital system and aspects related to adherence. Applicability burdens may either hinder or promote usage. The clinical importance will be evaluated in several domains, considering the primary endpoint, HRQoL, and secondary endpoints, including pain, nutrition, physical activity, sarcopenia, and fatigue. Consequently, comprehensive data collection and analysis will provide a deeper understanding of HRQoL determinants in the palliative care setting and broaden the evidence base to improve timely and cost-effective palliative care for PDAC patients. A key feature of the study design is that the digital approach will be integrated into standard clinical care and evaluated across three European sites.

To our knowledge, RELEVIUM-RCT is the first study to address typical critical requirements for comprehensive and holistic supportive care for PDAC patients regarding supportive care delivery, pain, nutrition, physical exercise, and cachexia. As recently reported by Bena Law et al. [73], supportive care must encompass and include measures to reduce pain, a multidisciplinary team and caregivers to affect the HRQoL, early assessment, and resistance exercise to tackle malnutrition and weight loss. Furthermore, RELEVIUM-RCT will indicate whether the participation of the PDAC patients and health professionals can increase through digital support as requested [29]. Generally, digital health solutions, including remote monitoring in supportive care, showed efficacy concerning fatigue, pain, HRQoL, and functional capacity [29]. Moreover, the system helps overcome the issues reported considering cancer care in rural areas [74].

Improvement in pain management is needed [28]. Thereby, patients should be able to document their symptoms electronically, and advanced comprehensive pain management strategies are essential components that require a substantial improvement in supportive and palliative care practices for PDAC patients [5]. A smartphone App with features similar to those of the RELEVIUM mobile App has been tested for pain management in a palliative setting in patients with solid tumors [75]. Patients who used the mobile App compared to standard therapy had a lower intensity of pain and a better perception of pain therapy. App use was also associated with a 40% reduction in hospital admissions. Furthermore, frequent reporting of symptoms to the oncology team through a web-based platform by patients with metastatic solid tumors compared to standard care resulted in significant prolonged survival in the patient-reported outcomes group [76].

Regarding nutrition, RELEVIUM-RCT partially contains measures comparable to the recently published study protocol STRONG (Support through Remote Observation and Nutrition Guidance) [77]. For example, screening for malnutrition through a web-based dashboard, monitoring dietary intake through a portable device, and evaluating the usability of the system, and patients' HRQoL. The advantage of RELEVIUM-RCT is that nutrition is embedded in a multimodal approach and interaction could be identified. After performing RELEVIUM-RCT, the results of the studies can be directly compared, since the time points for the evaluations after 4 and 8 weeks are identical [75].

RELEVIUM-RCT could demonstrate the effectiveness and safety of a method for remote monitoring exercise intensity and treatment in patients with PDAC who exercise without supervision. So far, research has been sparse on exploring these benefits for patients with PDAC. PDAC patients exercise at home during chemotherapy embedded in usual care improved dimensions within [78]. RELEVIUM-RCT builds on the approach of this study and combines exercise with measurements of nutrition, pain, and fatigue. Furthermore, the recent preliminary analysis, under the limitation of a small sample size, showed that during chemotherapy, a combined resistance and aerobic training program improves physical function and supports mental strength in older patients (60–75 years) [79]. In a prospective single-arm study, physical activity of PDAC patients was measured using wearable sensor technology for remote monitoring of daily activity and could supplement functional assessment and predict results [80]. Larger trials are needed to validate the findings, which have shown that fewer average step counts and poorer physical function scores were significantly associated with poor survival [80].

At the time of diagnosis, more than 85% of individuals with PDAC have cachexia, a condition associated with lower performance status, limited mobility, increased morbidity, and decreased survival rates [15]. Therefore, improving physical function through exercise during treatment is crucial to increase the tolerability of adjuvant or palliative therapies, reduce functional decline, and improve HRQoL before surgery or during recovery. However, the existing efficacy and guidelines for exercise prescription in the context of PDAC have yet to be determined [15]. Furthermore, the current literature shows a high prevalence of malnutrition in various cancers, including gastrointestinal cancers such as pancreatic cancer. Therefore, the combination of physical activity and nutritional support in RELEVIUM-RCT can partially reverse the complications associated with cachexia and malnutrition, according to [17].

The strengths of RELEVIUM-RCT reside primarily in its multifaceted scope, its multifaceted orientation, and the low-threshold accessibility of the system, along with its dense longitudinal data collection, which facilitates an effective exchange of information between the interdisciplinary treatment team and patients with their caregivers, ultimately helping in clinical decision-making processes. This intervention will employ a technology-driven strategy using an innovative technological solution that integrates wearable devices, sensors, and advanced algorithms for artificial intelligence, machine learning, and signal processing. This setup aims to provide a comprehensive understanding of the condition and actions of the patients, allowing physicians and caregivers to tailor their care plans to suit individual needs. If the study demonstrates that the benefits of the system exceed the associated costs, it could precipitate a novel, widespread digitalization of usual care thereby empowering patients and enabling clinicians to make more informed clinical decisions to enhance the HRQoL for patients. Therefore, the results of RELEVIUM-RCT will contribute data to the promising but relatively sparsely explored field of digital solutions to improve the HRQoL of cancer patients by integrating digital measures into standard clinical care, as determined in Ancona et al. (2025) [33].

A potential limitation or risk associated with the study is that its success is highly dependent on the dependability of both software and hardware and their consistent and reliable use. It is imperative that technical and clinical collaborators actively participate in study responsibilities and carry them out consistently. At the same time, patient engagement with the study and its technologies is critical to facilitate meaningful study results. This is particularly challenging in studies that involve lifestyle changes or changes in standard care practices. Furthermore, older adults may find it particularly challenging to navigate the technical tools or to be represented sufficiently in the sample, especially if they lack technological proficiency. However, specific criteria were incorporated into the study design to mitigate these challenges, as previously outlined in the discussion of the strengths of the study. The potentially high dropout rate should be acknowledged, and the possible loss of measurability of endpoints must be considered, as reported by Galvin et al. [80]. Furthermore, the ethical feasibility of conducting interventional research in patients with potentially short life expectancy should be carefully addressed. For example, the “EXPAN” trial protocol demonstrates that integrating supportive interventions (e.g. exercise) during neoadjuvant therapy is ethically acceptable and practically feasible [81].

The findings of our European RELEVIUM-RCT will provide comprehensive recommendations for the integration of this multimodal digital approach into palliative care. The recommendations will be addressed to the relevant authorities and discussed with leading national and European cancer organizations. In the future, in addition to studies on the necessary hallmarks of pancreatic cancer therapy as proposed by Halbrook et al. [2], the research could build on the results of RELEVIUM-RCT by developing further AI-supported algorithms based on multifaceted data considering all aspects of holistic, supportive therapy and empowerment of patients. Additionally, promising results recently published by Henderson et al. [82] on electronic cancer resilience evaluation, by Cobianchi et al. [83], by Mi et al. [84], and by Anderson et al. [85] on serious gaming should also be taken into account considering advanced digital support strategies for PDAC patients. Finally, RELEVIUM-RCT presents a complex multidisciplinary and, therefore, rare opportunity to examine the efficacy and usability of a holistic digital health approach integrated into regular care in three European countries for PDAC patients.

## Trial status

The trial, and therefore the recruitment of patients, is scheduled to be carried out in the second quarter of 2025 and should end in the first quarter of 2026.

## Data Availability

No datasets were generated or analysed during the current study.

## Abbreviations

6MWT: Six-Minute Walk Test
ACSM: American College of Sports Medicine
BBT: Brief-BESTest
BESTest: Brief Balance Evaluation Systems Test
BSFC-S: Burden Scale for Family Caregiver—Short Version
CRO: Contract Research Organization
DMC: Data monitoring committee
DMP: Data Management Plan
ECOG: Eastern Cooperative Oncology Group
eCRFs: Electronic Case Report Forms
EDA: Electrodermal activity
EDC: Electronic Data Capture
EORTC-QLQ-C30: European Organization for Research and Treatment of Cancer Quality of Life Questionnaire
ESPEN: European Society for Clinical Nutrition and Metabolism
FTSST: Five Times Sit to Stand Test
GCP: Good clinical practice
GDPR: General Data Protection Regulation
GLIM: Global Leadership Initiative on Malnutrition
HGS: Handgrip Strength
HGST: Handgrip Strength Test
HR: Heart rate
HRV: Heart rate variability
IPAQ-SF: International Physical Activity Questionnaire—Short Form
NRS: Numeric Rating Scale
PAR-Q +: Physical Activity Readiness Questionnaire
PDAC: Pancreatic Ductal Adenocarcinoma
PDI: Pain Disability Index
PERT check: Pancreatic Enzyme Replacement Therapy check
PG-SGA: Patient-Generated Subjective Global Assessment
PG-SGA-SF: PG-SGA-Short Form
HRQoL: Health-related Quality of Life
REDCap: Research Electronic Data Capture
RPE: Rate of Perceived Exertion
RPP: Rate of Perceived Pain
SARC-F: Strength Assistance (with Walking) Rising (from a Chair) Climbing (Stairs) Falls
SEFI: Self-Evaluation of Food Intake
TSC: Trial Steering committee
